# Urinary Proteomics Profiles Are Useful for Detection of Cancer Biomarkers and Changes Induced by Therapeutic Procedures

**DOI:** 10.3390/molecules24040794

**Published:** 2019-02-22

**Authors:** Emanuele Ferrari, Andrea Wittig, Fabrizio Basilico, Rossana Rossi, Antonella De Palma, Dario Di Silvestre, Wolfgang A.G. Sauerwein, Pier Luigi Mauri

**Affiliations:** 1 Proteomics and Metabolomics Unit, Institute for Biomedical Technologies (ITB-CNR), 20090 Segrate (MI), Italy; emanuele.ferrari@itb.cnr.it (E.F.); rossana.rossi@itb.cnr.it (R.R.); antonella.depalma@itb.cnr.it (A.D.P.); dario.disilvestre@itb.cnr.it (D.D.S.); 2 Dept. of Radiotherapy and Radiation Oncology, University Hospital Jena, 07743 Jena, Germany; andrea.wittig@med.uni-jena.de; 3 NCTeam, Strahlenklinik, Universitätsklinikum Essen, 45122 Essen, Germany; wolfgang.sauerwein@uni-due.de; 4 Istituto di Scienze della Vita, Scuola Superiore Sant’Anna, 56127 Pisa, Italy

**Keywords:** urine, thyroid cancer, squamous cell cancer of head and neck, BNCT, boron, proteomics, LC-MS, MudPIT

## Abstract

Boron neutron capture therapy (BNCT) is a binary cancer treatment modality where two different agents (^10^B and thermal neutrons) have to be present to produce an effect. A dedicated trial design is necessary for early clinical trials. The concentration of ^10^B in tissues is an accepted surrogate to predict BNCT effects on tissues. Tissue, blood, and urines were sampled after infusion of two different boron carriers, namely BSH and BPA in the frame of the European Organisation for Research and Treatment of Cancer (EORTC) trial 11001. In this study, urine samples were used to identify protein profiles prior and after drug infusion during surgery. Here, an approach that is based on the mass spectrometry (MS)-based proteomic analysis of urine samples from head and neck squamous cell carcinoma (HNSCC) and thyroid cancer patients is presented. This method allowed the identification of several inflammation- and cancer-related proteins, which could serve as tumor biomarkers. In addition, changes in the urinary proteome during and after therapeutic interventions were detected. In particular, a reduction of three proteins that were involved in inflammation has been observed: Galectin-3 Binding Protein, CD44, and osteopontin. The present work represents a proof of principle to follow proteasome changes during complex treatments based on urine samples.

## 1. Introduction

Boron neutron capture therapy (BNCT) is based on the high cross section of the non-radioactive isotope boron-10 for capturing thermal neutrons, leading to the nuclear reaction ^10^B(n,α)^7^Li [1]. The resulting high linear energy transfer (LET) particles have a very short range in tissues, limiting the damages to cells containing ^10^B [2]. For a successful tumor treatment, ^10^B has to be selectively delivered to tumor cells through specific boron-containing compounds. In the frame of the research project “Therapeutic strategies for Boron Neutron Capture Therapy (BNCT): Boron imaging” (financed by the European Commission QLK3-CT-1999-01067), several early clinical trials under the auspices of the European Organisation for Research and Treatment of Cancer (EORTC) were performed [3]. The investigation that is presented here is based on urine samples that were collected in the EORTC trial 11001 and urine samples from heavily smoking (>25 cigarettes/day) volunteers.

BNCT has already proven to be a promising tool in the treatment of a number of cancer, including head and neck squamous cell carcinoma (HNSCC) [4,5,6,7]. BNCT has been explored as an alternative therapy to the currently employed surgery, chemotherapy, or radiotherapy, bearing the advantages of less application (one or two doses) and the ability to maintain intact the oro-facial structures and functions [8]. Although no clinical application has been performed, BNCT has been discussed for the treatment of thyroid cancer in patients not responding to standard therapies [9,10].

To date, a very limited number of papers have investigated the molecular effects of boron compounds and BNCT. Molecular studies were mainly based on the monitoring of Boron-containing compounds in cell lines [11,12], tissue [13], plasma [14,15], and urine [16,17]. As far as we know, no extensive genomic or transcriptomic studies have been performed to evaluate the changes in the transcription that is induced by this treatment. A preliminary study focused on the DNA damage induced by irradiation while using a rat tumor graft model [18]; this work followed the levels of some proteins by means of western blotting and showed an upregulation of High mobility group box 1 (HMGB1), a nuclear protein involved in necrosis and inflammation processes, which was proposed as an early diagnostic marker. Surely, for the study of the effects of BNCT, proteomics is a promising tool, which has already been applied in an in vitro study on the effects of mercaptoundecahydrododecaborate (BSH) on phospholipid hydroperoxide glutathione peroxidase [19]; in this case, a gel-based approach was used to separate the native protein from the protein that was covalently bound to BSH, which were then characterized by means of liquid chromatography coupled to mass spectrometry (LC-MS). A more comprehensive application of the gel-based proteomics coupled to MALDI-TOF identification was reported in a study where a human oral squamous carcinoma cell line was treated with boronophenylalanine (BPA) and then irradiated with thermal neutrons [20]. Changes in the levels of 29 proteins were observed and they were mainly related to vesicle regulation, mRNA processing, and transcription.

In the last years, proteomics technologies considerably improved and a gel-free approach, which was mainly based on LC-MS (so called shotgun or MS-based proteomics), became the gold standard methodology to investigate the proteome profiles, increasing both the number of identified proteins and their quantitative analysis. In the present work, we took advantage of the availability of both urine samples, which were previously used for pharmacokinetic investigations, and of a gel- and label-free proteomics facility. We analyzed urine samples by means of multidimensional protein identification technology (MudPIT) to describe the urinary proteomes of HNSCC and thyroid cancer patients. Moreover, the effects on urinary proteome due to boron infusion (as BPA or BSH) in cancer patients were also investigated.

## 2. Results

### 2.1. Proteome Profiles of Urine

The multidimensional separation of peptides and the MS-based proteomic approach that was used in this work allowed for the characterization of proteome profiles of urine from healthy subjects and tumor patients. Thus, it was possible to compare healthy subjects and tumor patients with a simple and convenient shotgun protocol, which was based on liquid chromatography coupled to tandem mass spectrometry (LC-MS/MS). Good repeatability between technical replicates was observed (Figure S1). The complete list of proteins for each sample is reported in the supplementary materials (Table S1). When considering all of samples, 365 proteins were identified and 62.2% of them have more than one peptide. For the healthy group, 172 proteins were detected, while for HNSCC and thyroid cancer patients, 253 and 228 proteins were found, respectively. The three groups share 110 proteins, while the two groups of tumor patients share almost 60% of their proteins (Figure S2). An analysis of the Uniprot Keywords showed that 219 out of 365 proteins have a signal peptide sequence, indicating that they are destined to the endoplasmic reticulum and possibly secreted. When considering the groups separately, the analysis showed that about two-thirds of each group’s protein have a signal peptide. The Uniprot keywords also showed that 84 out of 365 proteins have a transmembrane domain, with no significant differences between healthy subjects and tumor patients. 

**Figure 1:**
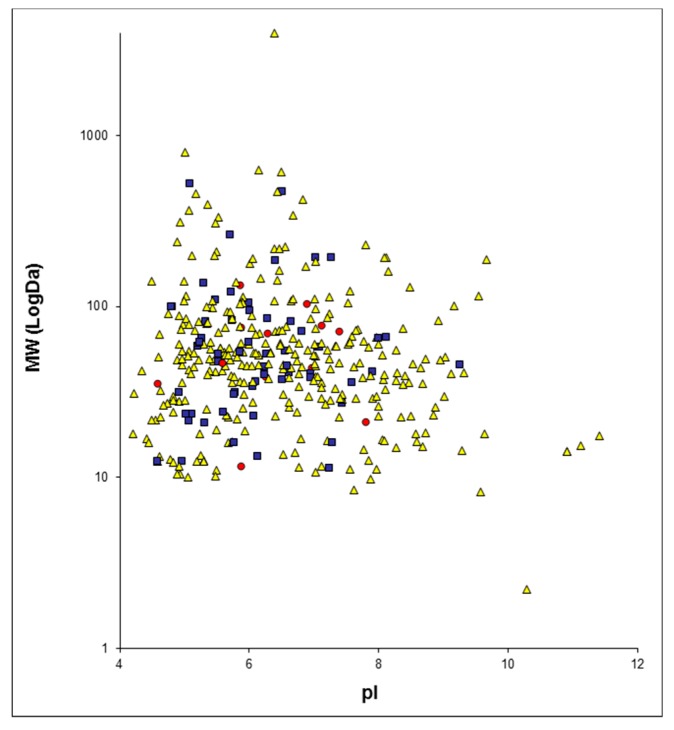
Two-dimensions map of the identified proteins from all analyzed urinary samples plotted with MAProMa software, according to their theoretical pI and MW (in Log scale). Proteins identified by 1 Peptide Spectrum Match (PSM) are reported as yellow triangles, those with PSMs between 2 and 4 as blue squares, and those with a number of PSMs over or equal to 5 as red circles.

The proteins identified for each group of patients were plotted on a two-dimensions map with MAProMa software, using theoretical molecular weight (MW) and isoelectrical point (pI) (Figures S3–S5). Figure 1 reports the two-dimensional map that was obtained by plotting all identified proteins. These maps show the ability of the shotgun approach to identify proteins in a wide range of MW and pI.

**Figure 2:**
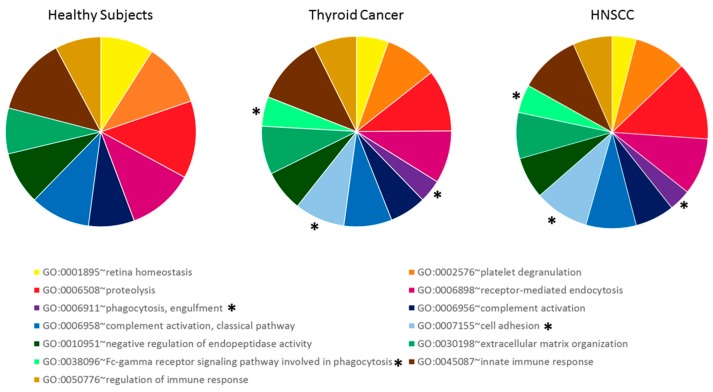
Enrichment in Gene Ontology (GO) biological process terms, using DAVID for each considered group. * denotes GO Terms enriched only in tumor patients’ proteome but not in healthy subjects.

An analysis of the enriched Gene Ontology (GO) biological process terms that was performed with DAVID confirms a qualitative similarity in the protein composition of urine between the three groups of subjects (Figure 2). In particular, the terms enriched in both tumor groups were the same, with little difference in the number of proteins for each term. It has to be noted that three of these terms were not found to be enriched in the urine of healthy subjects: phagocytosis, engulfment (GO:0006911), cell adhesion (GO:0007155), and Fc-gamma receptor signaling pathway that is involved in phagocytosis (GO:0038096). 

### 2.2. Differentially Secreted Proteins in Urine from Tumor Patients

**Figure 3:**
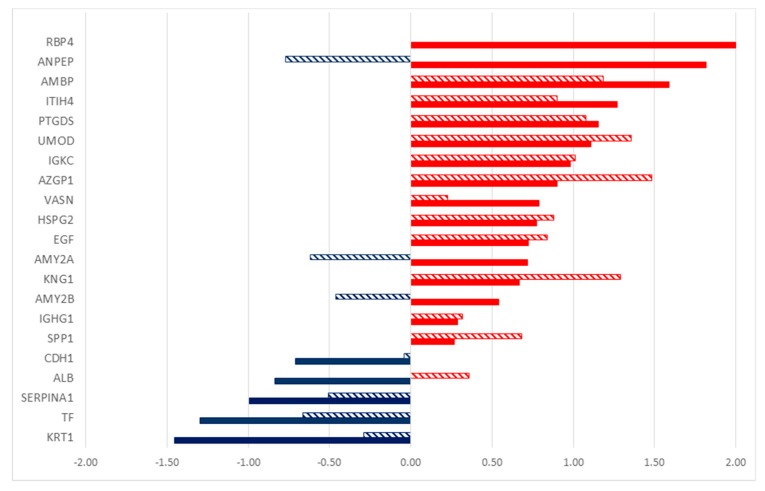
DAVE values of the significantly different levels of proteins found in urinary proteome of head and neck squamous cell carcinoma (HNSCC) patients (full bars) and thyroid cancer patients (dashed bars) in comparison to the healthy subjects. Red/positive and blue/negative bars correspond respectively to up- and down-secreted proteins in tumor patients compared to healthy subjects.

A quantitative approach was used, since there were no evident qualitative differences in the proteic composition of urine for the three groups. In this study, a label-free quantification has been applied, considering the total number of peptides (Peptide Spectrum Matches—PSMs, also called Spectral Count—SpC) for each protein. Indeed, this quantitative analysis led to the identification of significant alterations in the amount of single proteins that were secreted in the urine, when comparing healthy subjects with tumor patients, using DAVE and DCI algorithms [21]. Proteins with highly significant differences (i.e., |DAVE| > 0.2 and |DCI| > 10) are reported in Figure 3 and in Table S2, together with fold changes and G test values.

By comparing the healthy subjects with those that were affected by HNSCC, 14 proteins were found to be more abundant in urine from tumor patients, as indicated by the positive values of DAVE and DCI (Figure 3), while the levels of six proteins were higher in control subjects. Analogously, the analysis of urine from thyroid cancer patients led to the identification of 17 differentially secreted proteins in comparison to healthy volunteers. Among these proteins, 13 were overrepresented in affected subjects. It is interesting to note that the two types of tumors taken in account share similar highly secreted proteins, and some of them show significant differences in both cases. Nine proteins were higher in both tumors as compared to healthy patients, while three were lower: keratin, type II cytoskeletal 1 (KRT1), serotransferrin (TF), and alpha-1-antitrypsin (SERPINA1). The urinary proteins of the two tumor-affected groups were compared with the same method in order to identify the differences between them. Indeed, 21 proteins were differentially secreted, eight proteins were overrepresented in HNSCC patients, and 13 were overrepresented in thyroid cancer patients (Table S3).

**Figure 4:**
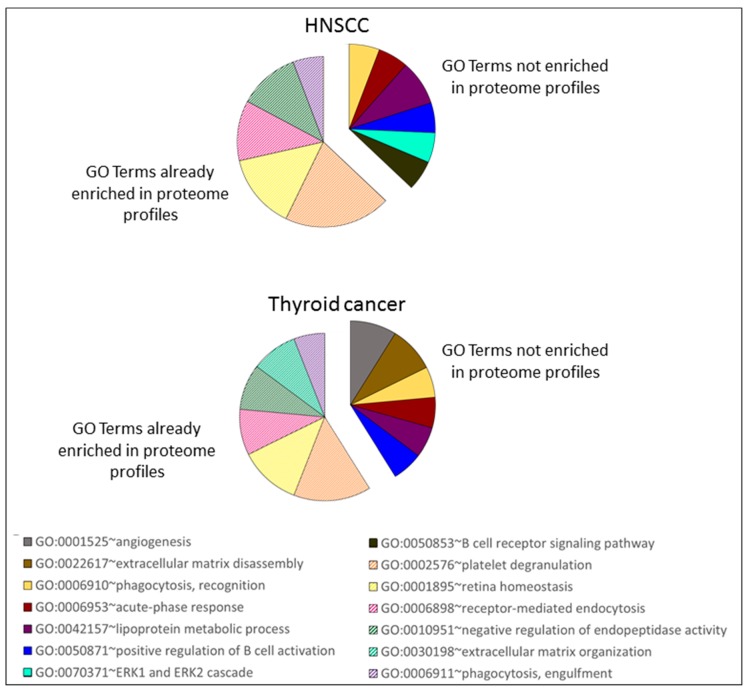
Enrichment in GO biological process terms, as performed with DAVID on the significantly different secreted proteins list reported in Table S2 and Figure 3 for HNSCC and thyroid cancer patients, as compared to healthy subjects. Each slice is proportional to the number of proteins identified for each GO biological process terms. The slices with the stripes pattern were already found to be enriched in the analysis that was performed on the total urinary proteome of HNSCC and thyroid cancer patients (see Figure 2).

In addition, the enriched GO biological process terms were searched for the proteins that were altered by the presence of tumor (Figure 4), and they showed similarity in the enriched terms between the two tumor entities. Only two terms were found to be enriched in HNSCC and not in thyroid cancer differentially secreted proteins: ERK1 and ERK2 cascade (GO:0070371) and B cell receptor signaling pathway (GO:0050853). Three terms were enriched exclusively in differentially secreted proteins of thyroid cancer patients: angiogenesis (GO:0001525), extracellular matrix disassembly (GO:0022617), and extracellular matrix organization (GO:0030198). A comparison with the GO term that was enriched in the whole urinary proteome profiles (Figure 2) showed that they share six GO biological process terms with the ones enriched in the differentially secreted proteins; these terms are reported in Figure 4 as slices with stripes pattern). The remaining terms were not enriched in urinary proteome profiles, and they are reported in Figure 4 with a colored solid fill: angiogenesis (GO:0001525), phagocytosis, recognition (GO:0006910), acute-phase response (GO:0006953), extracellular matrix disassembly (GO:0022617), lipoprotein metabolic process (GO:0042157), positive regulation of B cell activation (GO:0050871), ERK1 and ERK2 cascade (GO:0070371), and B cell receptor signaling pathway (GO:0050853). 

### 2.3. Proteome Profiles in Urine to Monitor Effects of Interventions 

#### 2.3.1. Infusion of a HNSCC Patient with BPA

**Figure 5:**
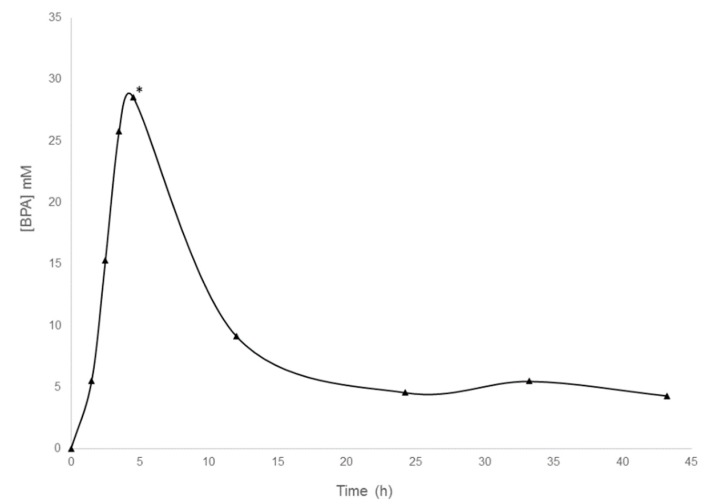
Concentration of boronophenylalanine (BPA) in the urine of the HNSCC patient analyzed. BPA infusion started at 0 min and ended at 1 h. * indicates the time point of urine sampling for proteomic analysis.

The first step in the BNCT therapy involves boron infusion before neutron irradiation; in this context, we investigated the effect of boron on urinary proteome. Specifically, the effect of the two boron-containing compounds BPA and BSH was investigated. One patient with a HNSCC was infused with BPA. Using flow-injection electrospray ionization mass spectrometry (FI-ESI-MS), the BPA-concentration in urine of the patient was measured before and after BPA-infusion (Figure 5) [16]. To evaluate the molecular effects of the compound on urine-secreted proteins, MudPIT proteomics analysis was performed on a urine sample that was taken before BPA-infusion and on a sample taken at the time point, when the urine concentration of BPA resulted in the highest. This time point equals 5 h after starting the BPA infusion, 6:45 h after starting anesthesia, and 6 h after starting the surgery (Figure 5).

**Figure 6:**
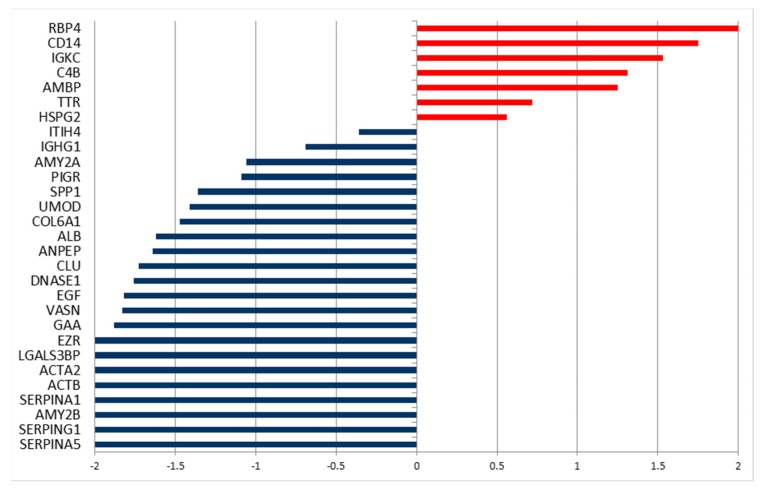
DAVE values of the significantly different secreted proteins that were found in urinary proteome of a patient with HNSCC, before and 5 h after the infusion of BPA and during surgery under anesthesia. The positive values indicate proteins increased after infusion, while negative values indicate proteins decreased after infusion.

This proteomic analysis allowed for a preliminary evaluation of the combined effects of BPA infusion during surgery under anesthesia on the urinary proteome. Significant changes in the levels of urinary proteins were identified using the MAProMa platform, which showed variations in the urinary level of 29 proteins (Figure 6 and Table S4). The majority (22) of protein levels decreased after BPA infusion (as indicated by negative values of DAVE and DCI). Among the proteins that decreased after the intervention were some that showed higher levels in the urine of HNSCC patients as compared to healthy subjects, namely Inter-alpha-trypsin inhibitor heavy chain H4 (ITIH4), Pancreatic alpha-amylase (AMY2A), osteopontin (abbreviated as OPN–gene name SPP1), uromodulin (UMOD), aminopeptidase N (ANPEP), pro-epidermal growth factor (EGF), and vasorin (VASN). Two proteins higher in the tumor patients, as compared to healthy volunteers, resulted in additional increase after the infusion of BPA: Retinol-binding protein 4 (RBP4) and Protein AMBP (AMBP). 

The GO analysis of enriched biological process terms (Table S5) showed that there are some terms that were affected by the infusion with BPA. Some of GO terms were found to be enriched only after infusion: positive regulation of protein ubiquitination involved in ubiquitin-dependent protein catabolic process (GO:2000060), leukocyte cell-cell adhesion (GO:0007159), retinol metabolic process (GO:0042572), retinoid metabolic process (GO:0001523), and carbohydrate metabolic process (GO:0005975). 

#### 2.3.2. Infusion of a Thyroid Cancer Patient with BSH

**Figure 7:**
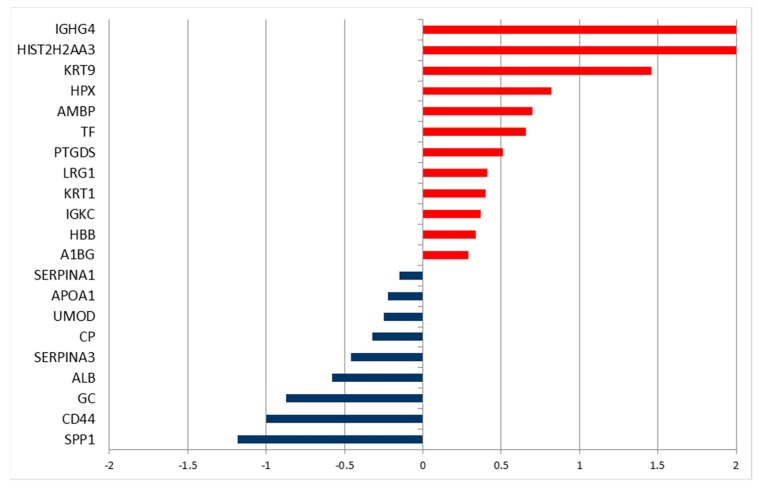
DAVE values of the significantly different secreted proteins found in urinary proteome of a patient affected by thyroid cancer, before and 10 h after the infusion with BSH and at the start of surgery under anesthesia. The positive values indicate proteins increased after infusion, while negative values indicate proteins decreased after infusion.

In a patient with thyroid cancer, we investigated the changes in the urinary proteome in urine samples that were taken before BSH infusion and 10 h after starting the infusion. This time point equals 30 min after starting anesthesia and just before starting surgery. Again, we identified the differential levels of proteins (Figure 7 and Table S6). Twenty-one proteins changed their levels in urine after BSH infusion. Only two proteins that were more abundant in the urine of tumor patients in comparison to healthy volunteers decreased: uromodulin and OPN. Interestingly, there was an increase of serotransferrin (TF) after BSH infusion, while levels of this protein were lower in tumor patients as compared to healthy subjects.

For this patient, a total number of 24 GO biological process terms were enriched, when considering the differentially secreted proteins after BSH-infusion (Table S7). Eleven GO terms were specifically enriched in relation to BSH infusion: Defense response to bacterium (GO:0042742), inflammatory response (GO:0006954), phagocytosis, engulfment (GO:0006911), B cell receptor signaling pathway (GO:0050853), cellular oxidant detoxification (GO:0098869), cellular iron ion homeostasis (GO:0006879), vitamin transport (GO:0051180), oxygen transport (GO:0015671), establishment of skin barrier (GO:0061436), hydrogen peroxide catabolic process (GO:0042744), and positive regulation of cell death (GO:0010942).

### 2.4. Reduction of Proteins Related to Inflammation after Infusion: galectin 3 Binding Protein, CD44, and OPN

**Figure 8:**
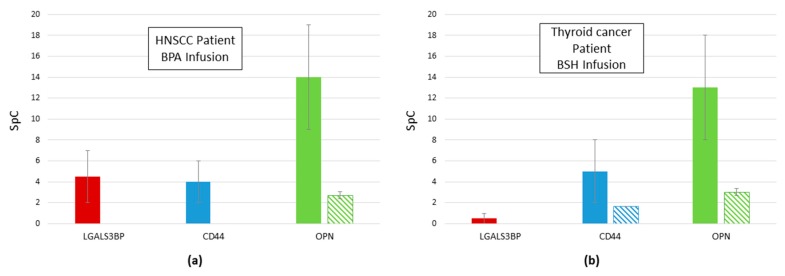
(**a**) Total amount of LGALS3BP (red fill), CD44 (blue fill), and osteopontin (OPN) (SPP1, green fill) found in urine of the HNSCC patient infused with BPA before (solid fill) and 5 h after infusion (diagonal lines fill). (**b**) Total amount of LGALS3BP (red fill), CD44 (blue fill), and OPN (SPP1, green fill) found in urine of the thyroid cancer patient infused with BSH before (solid fill) and 10 h after infusion (diagonal lines fill). Data are expressed as SpC ± SEM.

In both treated patients, regardless of the boron compound infused, a reduction in three proteins has been observed: galectin 3 Binding Protein (LGALS3BP), CD44, and OPN (SPP1) (Figure 8). These glycoproteins are involved in inflammatory processes and possibly tumor progression [22,23,24]. All three proteins were reduced or even absent in urine that was sampled after boron compound infusion and during anesthesia and surgery.

## 3. Discussion

In this work, we present the potential of LC-MS-based proteomics for monitoring treatment, using an easily collectable biological fluid, such as urine. Protein profiles from urine samples allowed for the discovery of potential tumor biomarkers by using a label-free quantitative comparison between healthy subjects and HNSCC or thyroid cancer patients to find proteins that were differentially secreted in urine from tumor patients. The urinary proteomes of cancer patients showed similarity in their overall composition, as confirmed by the GO biological process enriched terms. In particular, “cell adhesion” term was found to be enriched in patients but not in healthy volunteers, suggesting that this biological process is disrupted by the presence of tumors and it is reflected in urine protein composition. 

In both groups of patients with tumors, a great increase in uromodulin (UMOD) is interesting to note, as this is known to be the most abundant in urine [25], as confirmed in our data for all subjects. Although the total amount of uromodulin is related to the disease, it is hard to explain this increase with respect to the healthy subjects, since the role of uromodulin is still not completely clear in both physiological and pathological conditions. It is possible that uromodulin is related to an inflammatory state that is observed in tumor patients, since its involvement in inflammatory processes in kidney injury has been demonstrated [26].

Indeed, some of the proteins with higher levels in both groups are involved in inflammatory processes. Among these, one of the most interesting is ITIH4, which is involved in inflammatory responses to trauma [27]. Several groups have also demonstrated the potential of ITIH4 as a predictive biomarker in a number of tumor entities [28,29,30]. In our investigation, the ITIH4 levels are elevated in the urine of patients with HNSCC and in patients with thyroid cancer, but it is significantly higher in the urine of HNSCC patients as compared to the urine of thyroid cancer patients. 

Two immunoglobulins were found to be excreted in urine from tumor-affected subjects to a higher extent, with no significant differences between the two tumor entities: Ig gamma-1 chain C region (IGHG1), and Ig kappa chain C region (IGKC). In particular, IGKC show a very distinctive increase, which was similar for both of the tumor entities examined. Proteins that are secreted in urine clearly reflect what happens in the organism and, in particular, the inflammation processes in progress. OPN (SPP1) is an interesting protein that is involved in inflammatory processes and it resulted higher in urine samples of tumor patients. Many cells, including the cells of the immune system but also tumor cells, secrete it. Specifically, OPN increases in breast, colon, gastric, liver, lung, and prostate cancers [31]. It interacts with the CD44 receptor and it is considered to be one of the most promising prognostic biomarkers for cancer [32]. 

The presence of inflammatory processes in tumor patient seems to be also confirmed by the GO biological process enrichment analysis of differentially secreted proteins. Indeed, the enrichment of GO terms, such as acute-phase response (GO:0006953), positive regulation of B cell activation (GO:0050871), and B cell receptor signaling pathway (GO:0050853), indicate that the inflammatory processes are in progress and they are reflected by changes in urine proteome composition. 

Obviously, inflammation-related proteins are not sufficient for the diagnosis of cancer, since they could be linked to additional conditions. Indeed, among the other proteins found to be excreted to an enhanced extent, some have been already related to cancer and could therefore be considered to be cancer biomarkers. As an example, aminopeptidase N (ANPEP) has been associated with the growth of different human cancers and has been suggested as a suitable target for antineoplastic treatment approaches [33]. In particular, an increase in aminopeptidase N activity in HNSCC has already been observed [34]. Vasorin (VASN) is a type I transmembrane protein that plays an important role in tumor development and vasculogenesis; it is a potential serum biomarker of hepatocellular carcinoma and it may be a drug target for its treatment [35]. Pancreatic alpha-amylase (AMY2A) has been related to cell adhesion, growth, and the invasion of cancer cells and it has been proposed as a candidate urine biomarker for pancreatic cancer [36]. Zinc-alpha-2-glycoprotein (AZGP1) is a protein that is known to have a role in different tumor entities. An overexpression of zinc-alpha-2-glycoprotein has been observed in a proteomic analysis of saliva and serum from patients that are diagnosed with HNSCC [37]. 

Some of the proteins that were identified as significantly higher in the urine of tumor patients as compared to healthy subjects have been already proposed as possible biomarkers, although no cancer-related functions have been identified so far. For example, the protein AMBP (AMBP), which shows high levels in urine from patients with both HNSCC and thyroid cancer, has been found to also be secreted in urine from the bladder [38] and prostate cancer patients [39] and in other biofluids (such as cerebrospinal fluid and serum) of such patients [40,41]. 

The effect of tumor could also lead to a loss of function and therefore to a decrease in particular proteins. As an example, the reduction in serotransferrin (TF) observed is possibly related to a dysfunction in iron homeostasis. This could lead to further damage of the tissues following cancer progression. Additionally, the reduction of cadherin 1 (CDH1), a growth and invasion suppressor, is an interesting observation [42].

These data show that LC-MS proteomic analysis of urine could be a suitable and convenient method for the characterization of different types of cancer. In particular, it is possible to simultaneously follow several urine-secreted proteins that were altered by the presence of a tumor, thus creating a tool more reliable than an approach based on the changes of exclusively one single protein. Furthermore, specific proteins in urine could be followed for differentiating one cancer from another. As an example, in this study, we showed that retinol-binding protein 4 (RBP4) was exclusively found in the urine of patients with HNSCC but not thyroid cancer, indicating that this protein could be a specific HNSCC biomarker. RBP4 is an adipokine that is mainly produced by adipose tissue and its serum levels in medullary thyroid carcinoma patients are not significantly different from those that are found in healthy individuals [43]. On the contrary, RBP4 was recently reported as a potential biomarker of oral squamous cell carcinoma, in combination with clusterin, haptoglobin, complement C3c, and proapolipoprotein A1 [44].

The method presented has been proven to also be useful for monitoring the effects at a molecular level of two different boron compounds, used for BNCT: BPA and BSH. The infusion of patients with either one of the boron compounds induces changes in the proteins that were secreted in the urine. In particular, we observed a reduction in proteins that were involved in inflammation, such as GALS3BP and CD44. Additionally, OPN was decreased after boron infusion, both by BPA and BSH. OPN is a secreted protein that is overexpressed in several human carcinomas. It has been implicated in different mechanisms of tumor progression: cell proliferation, survival, drug resistance, invasion through its role in intercellular communication, and tumor microenvironment formation [45]. Moreover, high levels of OPN have been found in cerebrospinal fluid and serum of cancer patients [46] and it is considered a possible target for cancer therapy [23]. It is to be noted that the role in inflammation of OPN is possibly mediated by CD44 isoforms [47,48], suggesting that a specific inflammatory pathway could be the target of BSH or BPA. 

It has already been reported that other boron-containing compounds are able to reduce inflammation both in animals and in humans [49], suggesting a beneficial effect of boron as a regulator of the inflammatory reactions with a possible role in cancer and other diseases [50]. Our proteomics data suggest that BPA and BSH could also reduce tumor-derived inflammation, thus possibly helping the treatment. Certainly, further experiments are needed to confirm this hypothesis and to find other inflammation-related proteins that are reduced by boron infusion. Of note, in the setting of this feasibility investigation, the effects of drugs infused for anesthesia, and/or surgery itself might influence the proteasome of the urine as well; thus, the observed effects cannot be solely attributed to the boron compounds.

To date, very few studies investigated the effects of BNCT on the proteome. Although BNCT is a binary therapy, up to now, the existing studies analyze the effects of both boron infusion and neutron irradiation, but not the effect of either component of this treatment modality. In very few cases, the effect of neutron irradiation with and without the infusion of a boron compound were evaluated, such as the unique proteomics investigation after BNCT, as performed by Sato et al. [20]. Unfortunately, their data cannot be compared with those that were obtained in the present work, because the authors analyzed squamous cell carcinoma SAS cells in vitro irradiated with a thermal neutron beam with and without BPA. The authors used a 2D gel approach and characterized approximately 20 proteins that were mainly related to the endoplasmic reticulum-localized lymphoid-restricted protein.

The results of our study demonstrate the ability of shotgun proteomics based on the MudPIT approach to identify a great number of potential protein biomarkers in urine. This is of growing interest, since it is becoming clear that urinary proteome reflects changes that are induced by several diseases, including different types of cancer. Furthermore, urine could be preferred to blood or cerebrospinal fluid because of the easier and less invasive procedure of collection. The presented data must be considered as a feasibility evaluation due to low patients’ numbers and urine samples that are not systematically drawn for this purpose. Obviously, larger patient sets will also help to discover the influence on the urinary proteome of other factors, such as age, gender, medication, or diet. The proteomic analysis of urine is non-invasive and fast method, not only for tumor diagnosis, but also for following the effects of treatment at the molecular level. Indeed, in the future, urine and possibly other biofluids will be analyzed during and after the treatment in order to follow and possibly verify the changes that are induced by therapy. In addition, urinary exosomes will be studied, since it seems that biomarkers are enriched in secreted vesicles, facilitating the identification of disease-specific proteins [51]. A forthcoming application of this approach could also be the identification of biomarkers that are useful in monitoring the BNCT treatment and potentially predicting the response of patients to treatment, allowing for the application of the best therapy for each person in the pursuit of personalized medicine. 

Finally, although, in BNCT trials, the molecular effects due to boron-containing compounds were never systematically evaluated, our data indicate the proteome analysis of urine samples as a specific and informative method, and it may be a potential mirror of disease. These results suggest that future investigations with this method should evaluate the effects of boron compound infusion. The present work represents a proof of principle to investigate the BNCT treatment effects; of course, presented data should be considered as a preliminary evaluation and they need to be confirmed by further analyses on larger patient sets through a multicenter collaboration.

## 4. Materials and Methods 

### 4.1. Patients Recruitment and Urine Collection

The samples were collected from patients who participated in the clinical trial EORTC 11001, “^10^B-uptake in different tumors using the boron compounds BSH and BPA”. This study investigates the delivery of ^10^B to certain tumor entities by the boron compounds BSH and BPA. The study procedures included intravenous infusion of BSH and BPA prior to a planned surgical procedure that was necessary for tumor treatment and sample collection, but no radiotherapy with BNCT. All of the participants gave written informed consent prior to inclusion. The evaluation presented here became possible because all of the participants agreed that the samples taken might also be investigated by innovative procedures that were not yet available at the time of the sampling. The Protocol Review Committee of the EORTC and the Ethics Committee of the Medical Faculty of the University Duisburg-Essen approved the trial. The complete clinical characteristics of patients are reported in Table S8.

In addition to sample collection within the EORTC trial 11001, urine samples were collected after written informed consent from heavily smoking (>25 cigarettes/day) healthy staff members of University Hospital Essen, who volunteered to participate. Healthy volunteers did not receive any medication or study the compounds. The age of the healthy volunteers were in the same range of the tumor-affected subjects. 

All of the analyzed samples from cancer patients are reported in Table 1.

### 4.2. Boron Compounds and Urine Collection

BSH and BPA were purchased from KATCHEM Ltd. (Praha, Czech Republic). The quality of the study medication was strictly controlled, including an examination of the identity of compounds by infra-red spectroscopy, monitoring of purity by high pressure liquid chromatography, and test for pyrogenicity. The enrichment of ^10^B was ≥99.6% in both compounds and it was tested with prompt gamma ray spectroscopy (PGRA) and inductively coupled plasma-atomic emission spectroscopy (ICP-AES). The injection-solutions were prepared according to standard operating procedures that were established for the EORTC trials 11961, 11001, and 11011 [52,53]. 

To improve solubility, BPA (100 mg/kg) was infused as a BPA–fructose complex, within 1 h. Urine samples were taken before and up to 34 h after the start of infusion. Urine samples were immediately frozen at −20 °C until analysis. 

BSH (50 mg/kg) was dissolved in saline and infused within 1 h. The urine samples were taken before the start of infusion and at 9.5 h after the start of the BSH infusion. Samples were immediately frozen at −20 °C until analysis.

In the past, urine samples from patients that were infused by boron-containing compounds were investigated to determine compound pharmacokinetics [16]. We took advantages of the availability of these samples and the shotgun proteomics approach to describe the related proteome profiles. Specifically, urine samples from four patients with HNSCC and three patients with thyroid cancer, which were collected before infusion of the boron-containing compounds, were analyzed and compared to the healthy volunteers. Urine samples from two patients (one patient with HNSCC and one patient with thyroid cancer) were analyzed before compound infusion and after compound infusion in order to evaluate the eventual changes in the urinary proteome that is caused by the infusion of the boron-containing drugs BPA and BSH, as well as surgery and anesthesia.

### 4.3. Urine Preparation for Proteomics Analysis

For each sample, 600 µL of urine were centrifuged at 2000 g for 10 min. Supernatant was collected and filtered twice with the Microcon YM10 system to eliminate salts. The protein concentration was assayed using the SPN^TM^–Protein Assay kit (G-Biosciences, St. Louis, MO, USA).

### 4.4. Enzymatic Digestion of Protein Samples

Sequencing grade modified trypsin (Promega, Madison, WI, USA) was added to 50 µL of conditioned medium containing 1 µg protein at a 1:50 enzyme:protein ratio (*w:w*) in 100 mM ammonium bicarbonate, pH 8.0, and then incubated at 37 °C overnight. The reaction was stopped by acidification with trifluoroacetic acid. A second aliquot of each sample was digested with pepsin (Sigma-Aldrich, Milan, Italy) at 1:50 enzyme:protein ratio (*w:w*) in 100 mM ammonium acetate pH 3.0 at room temperature for 4 h and then immediately analyzed. Ten microliters of the peptide mixture were directly injected into the 2DC-MS/MS.

### 4.5. Two-Dimensional Capillary Chromatography-Tandem Mass Spectrometry (2DC-MS/MS) Analysis

Ten microliters of the peptide mixtures, as obtained from the digestion of the protein samples, were analyzed by means of two-dimensional microchromatography coupled with an ion trap mass spectrometer, using the ProteomeX system (ThermoElectron, San Josè, CA, USA) that was equipped with Bioworks 3.1 as graphical interface for data handling. Peptide mixtures were first separated by means of strong cation-exchange chromatography (Biobasic-SCX column, 5 µm, 0.3 ID × 150 mm, ThermoHypersil, Bellofonte, PA, USA) using seven steps of increasing ammonium chloride concentration (0, 50, 100, 150, 200, 300, and 600 mM). Each salt step was directly loaded onto the reversed phase column (Biobasic-C18, 0.180 ID × 100 mm, ThermoHypersil, Bellofonte, PA) and then separated with an acetonitrile gradient: eluent A, 0.1% formic acid in water; eluent B, 0.1% formic acid in acetonitrile; the gradient profile was 5% B for 3 min, followed by 5 to 50% B within 40 min. Peptides that were eluted from the C18 column were sent directly to an ion trap LCQXP mass spectrometer through an ESI ion source interface that was equipped with a metal needle (10 µm ID). The heated capillary was held at 160 °C, ion spray 3.2 kV, and capillary voltage 67 V. Spectra were acquired in positive mode (in the range of 400–1600 *m/z*) using dynamic exclusion for MS/MS analysis (collision energy 35%).

### 4.6. Data Processing of MS Results

All data generated were searched using the Sequest HT search engine contained in the Proteome Discoverer software, version 2.1 (Thermo Scientific, Waltham, MA, USA). The experimental MS/MS spectra were correlated to tryptic peptide sequences by comparison with the theoretical mass spectra that were obtained by in silico digestion of the Uniprot human proteome database (70,726 entries), downloaded in January 2017 (www.uniprot.org). The following criteria were used for the identification of peptide sequences and related proteins: trypsin as enzyme, three missed cleavages per peptide, mass tolerances of ±1 Da for precursor ions, and ±1 Da for fragment ions. Percolator node was used with a target-decoy strategy to give final false discovery rates (FDR) at Peptide Spectrum Match (PSM) level of 0.01 (strict) based on q-values, when considering a maximum deltaCN of 0.05 [54]. Only peptides with high confidence, minimum peptide length of six amino acids, and rank 1 were considered. Protein grouping and strict parsimony principle were applied. The MS data have been deposited to the ProteomeXchange Consortium via the PRIDE partner repository [55] and they are available upon request to the corresponding author.

The output data that were obtained from SEQUEST software were treated with the MAProMa (Multidimensional Algorithm Protein Map) in-house algorithm for a comparison of the protein lists, evaluation of relative abundances, and plotting virtual 2D maps [56]. A label-free approach has been used, counting the peptide spectrum matches (PSMs) for each identified protein [21]. Fold changes were calculated as natural logarithm of the SpC (SpC1/SpC2) [57]; the natural logarithm of the proteins identified exclusively in one of the two compared groups was set to ±100. G test was performed for the differentially expressed protein, as already reported for label-free quantification [58].

Venn diagrams were created with Venny (http://bioinfogp.cnb.csic.es/tools/venny/index.html).

### 4.7. GO Terms Enrichment

GO terms enrichment was performed with DAVID 6.8 (https://david.ncifcrf.gov/), using Uniprot accessions [59]. Biological process terms were considered to be enriched when the p-value and Bonferroni test results were below 0.0001 and at least 10 gene were found for the term. 

## Figures and Tables

**Table 1:** Analyzed cancer patients’ age at the time of urine collection and tumor diagnosis.

Subject	Age	Diagnosis
P12	45.3	Squamous cell carcinoma of the head and neck region
P21	50.6	Squamous cell carcinoma of the head and neck region
P20	68.5	Squamous cell carcinoma of the head and neck region
P22	53.1	Squamous cell carcinoma of the head and neck region
P11	79.6	Thyroid carcinoma
P14	34.6	Papillary thyroid carcinoma
P16	34.0	Papillary thyroid carcinoma

## Supplementary Materials

The following are available online. Figure S1: Comparison between two different replicates of patient 20, Figure S2: Two dimensions map of healthy subjects urine proteome profiles, Figure S3: Two dimensions map of HNSCC patients urine proteome profiles, Figure S4: Two dimensions map of thyroid cancer patients urine proteome profiles, Figure S5: Venn diagram of proteins distribution among the three groups, Table S1: List of all proteins identified in the samples analyzed, Table S2: DAVE and DCI values, fold changes, and G test values of the proteins differentially secreted in urine from healthy subjects versus those from cancer patients, Table S3: DAVE and DCI values, fold changes, and G test values of the proteins differentially secreted in urine from thyroid cancer patients versus those from HNSCC patients, Table S4: DAVE and DCI values of the proteins differentially secreted in urine from a patient affected by HNSCC, before and 5 h after the infusion with BPA, Table S5: GO biological process terms enriched in differentially expressed proteins for HNSCC patient treated with BPA, Table S6: DAVE and DCI values of the proteins differentially secreted in urine from a patient affected by thyroid cancer, before and 4 h after the infusion with BSH, Table S7: GO biological process terms enriched in differentially expressed proteins for thyroid cancer patient treated with BSH, Table S8: Subjects analyzed with complete clinical characteristics.
