# Supplementary material for: Urinary Proteomics Profiles Are Useful for Detection of Cancer Biomarkers and Changes Induced by Therapeutic Procedures
Source: Molecules. 2019 Feb 22;24(4):794. doi: 10.3390/molecules24040794 (PMC6412696; doi:10.3390/molecules24040794)
Supplement: Supplementary file 1 [file molecules-24-00794-s001.zip › Table S4.docx]

| **Protein name** | **Gene Name** | **Accession** | **DAVE**  **After vs before** | **DCI**  **After vs before** |
| --- | --- | --- | --- | --- |
| Retinol-binding protein 4 | RBP4 | P02753 | 2.00 | 481 |
| Monocyte differentiation antigen CD14 | CD14 | P08571 | 1.75 | 28 |
| Ig kappa chain C region | IGKC | P01834 | 1.53 | 332 |
| Complement C4-B | C4B | P0C0L5 | 1.31 | 11 |
| Protein AMBP | AMBP | P02760 | 1.25 | 265 |
| Transthyretin | TTR | P02766 | 0.72 | 11 |
| Basement membrane-specific heparan sulfate proteoglycan core protein | HSPG2 | P98160 | 0.56 | 39 |
| ITIH4 protein | ITIH4 | B7ZKJ8 | -0.36 | -28 |
| Ig gamma-1 chain C region (Fragment) | IGHG1 | A0A0A0MS08 | -0.69 | -16 |
| Pancreatic alpha-amylase | AMY2A | P04746 | -1.06 | -65 |
| Polymeric immunoglobulin receptor | PIGR | P01833 | -1.09 | -33 |
| Osteopontin | SPP1 | P10451 | -1.36 | -94 |
| Uromodulin | UMOD | X6RBG4 | -1.41 | **-257** |
| Collagen alpha-1(VI) chain | COL6A1 | P12109 | -1.47 | -15 |
| Serum albumin | ALB | P02768 | -1.62 | -60 |
| Aminopeptidase N | ANPEP | P15144 | -1.64 | -143 |
| Clusterin | CLU | P10909 | -1.73 | -24 |
| Deoxyribonuclease-1 | DNASE1 | P24855 | -1.76 | -32 |
| Pro-epidermal growth factor | EGF | P01133 | -1.82 | -55 |
| Vasorin | VASN | Q6EMK4 | -1.83 | -66 |
| Lysosomal alpha-glucosidase | GAA | P10253 | -1.88 | -60 |
| Plasma serine protease inhibitor | SERPINA5 | P05154 | -2.00 | -45 |
| Plasma protease C1 inhibitor | SERPING1 | P05155 | -2.00 | -36 |
| Alpha-amylase 2B | AMY2B | P19961 | -2.00 | -32 |
| Alpha-1-antitrypsin | SERPINA1 | P01009 | -2.00 | -25 |
| Actin, cytoplasmic 1 | ACTB | P60709 | -2.00 | -21 |
| Actin, aortic smooth muscle | ACTA2 | P62736 | -2.00 | -13 |
| Galectin-3-binding protein | LGALS3BP | Q08380 | -2.00 | -10 |
| Ezrin | EZR | E7EQR4 | -2.00 | -10 |

**Table S4.** DAVE and DCI values of the proteins differentially secreted in urine from a patient affected by HNSCC, before and 5 h after the infusion with BPA. The positive values indicate proteins increased after infusion, while negative values indicate proteins decreased after infusion.
