# Supplementary material for: Urinary Proteomics Profiles Are Useful for Detection of Cancer Biomarkers and Changes Induced by Therapeutic Procedures
Source: Molecules. 2019 Feb 22;24(4):794. doi: 10.3390/molecules24040794 (PMC6412696; doi:10.3390/molecules24040794)
Supplement: Supplementary file 1 [file molecules-24-00794-s001.zip › Table S2.docx]

| **Protein name** | **Gene Name** | **Accession** | **DAVE**  **HNSCC vs HS** | **DCI**  **HNSCC vs HS** | **Fold Change**  **HNSCC vs HS** | **G test HNSCC vs HS** | **DAVE**  **TC vs HS** | **DCI**  **TC vs HS** | **Fold Change**  **TC vs HS** | **G test**  **TC vs HS** |
| --- | --- | --- | --- | --- | --- | --- | --- | --- | --- | --- |
| Cadherin-1 | CDH1 | A0A087WXI5 | -0.71 | -12 | -0.74 | 1.0* | -0.04* | -1* | -0.04 | 0.0* |
| Ig gamma-1 chain C region (Fragment) | IGHG1 | A0A0A0MS08 | 0.29 | 11 | 0.29 | 0.3* | 0.32 | 12 | 0.32 | 0.3* |
| ITIH4 protein | ITIH4 | B7ZKJ8 | 1.27 | 39 | 1.50 | 4.8 | 0.90 | 12 | 0.97 | 1.5* |
| Alpha-1-antitrypsin | SERPINA1 | P01009 | -1.00 | -180 | -1.09 | 7.0 | -0.51 | -131 | -0.52 | 2.1* |
| Kininogen-1 | KNG1 | P01042 | 0.67* | 3* | 0.69 | 0.5* | 1.29 | 19 | 1.54 | 3.5* |
| Pro-epidermal growth factor | EGF | P01133 | 0.72 | 13 | 0.76 | 1.2* | 0.84 | 19 | 0.89 | 1.7* |
| Ig kappa chain C region | IGKC | P01834 | 0.98 | 99 | 1.07 | 5.1 | 1.01 | 109 | 1.11 | 5.6 |
| Apolipoprotein A-I | APOA1 | P02647 | -1.50* | -2* | -1.95 | 1.3* | 0.98 | 12 | 1.07 | 1.7* |
| Retinol-binding protein 4 | RBP4 | P02753 | 2.00 | 18 | 100.00 | 8.3 | - | - | - | 0.0* |
| Protein AMBP | AMBP | P02760 | 1.59 | 169 | 2.16 | 14.9 | 1.18 | 32 | 1.36 | 3.9 |
| Serum albumin | ALB | P02768 | -0.83 | **-297** | -0.89 | 6.8 | 0.36 | 379 | 0.36 | 2.1* |
| Serotransferrin | TF | P02787 | -1.30 | -66 | -1.55 | 6.5 | -0.66 | -52 | -0.69 | 2.0* |
| Keratin, type II cytoskeletal 1 | KRT1 | P04264 | -1.45 | -25 | -1.85 | 4.9 | -0.29 | -11 | -0.29 | 0.3* |
| Pancreatic alpha-amylase | AMY2A | P04746 | 0.72 | 28 | 0.75 | 1.7* | -0.62* | -6* | -0.64 | 0.6* |
| Osteopontin | SPP1 | P10451 | 0.27 | 11 | 0.27 | 0.2* | 0.68 | 50 | 0.71 | 2.0* |
| Aminopeptidase N | ANPEP | P15144 | 1.82 | 14 | 3.04 | 5.6 | -0.77* | -0* | -0.81 | 0.1* |
| Alpha-amylase 2B | AMY2B | P19961 | 0.54 | 12 | 0.55 | 0.7* | -0.46* | -3* | -0.47 | 0.3* |
| Zinc-alpha-2-glycoprotein | AZGP1 | P25311 | 0.90* | 6* | 0.97 | 1.0* | 1.48 | 42 | 1.91 | 6.6 |
| Prostaglandin-H2 D-isomerase | PTGDS | P41222 | 1.15 | 33 | 1.31 | 3.8 | 1.08 | 26 | 1.20 | 3.0* |
| Basement membrane-specific heparan sulfate proteoglycan core protein | HSPG2 | P98160 | 0.77 | 12 | 0.82 | 1.2* | 0.88 | 16 | 0.95 | 1.7* |
| Vasorin | VASN | Q6EMK4 | 0.79 | 29 | 0.84 | 1.9* | 0.23* | 4* | 0.23 | 0.1* |
| Uromodulin | UMOD | X6RBG4 | 1.11 | 235 | 1.25 | 9.5 | 1.36 | 553 | 1.65 | 20.4 |

**Table S2.** DAVE and DCI values of the proteins differentially secreted in urine from healthy subjects versus those from cancer patients. The positive values indicate proteins overrepresented in cancer patients, while negative values indicate proteins overrepresented in healthy subjects. Fold change is expressed as LN(SpC_HNSCC_/SpC_HS_) or LN(SpC_TC_/SpC_HS_). For G-test a value > 3.8 is equivalent to a p-value <0.05, while a G test value >11.2 is equivalent to a p-value <0.01 * denotes non-significant changes.
