# Supplementary material for: Urinary Proteomics Profiles Are Useful for Detection of Cancer Biomarkers and Changes Induced by Therapeutic Procedures
Source: Molecules. 2019 Feb 22;24(4):794. doi: 10.3390/molecules24040794 (PMC6412696; doi:10.3390/molecules24040794)
Supplement: Supplementary file 1 [file molecules-24-00794-s001.zip › Supplementary Figures captions.docx]

**Supplementary Figures captions**

**Figure S1.** Comparison between two different replicates of patient 20. Each axis report the PSMs for each protein found in the technical replicate.

**Figure S2.** Two dimensions map of healthy subjects urine proteome profiles plotted with MAProMa software. Proteins are plotted according to their theoretical pI and MW (in Log scale). Protein with 1 PSM are reported as yellow triangles, those with PSMs between 2 and 4 as blue squares, and those with a number of PSMs over or equal to 5 as red circles.

**Figure S3.** Two dimensions map of HNSCC patients urine proteome profiles plotted with MAProMa software. Proteins are plotted according to their theoretical pI and MW (in Log scale). Protein with 1 PSM are reported as yellow triangles, those with PSMs between 2 and 4 as blue squares, and those with a number of PSMs over or equal to 5 as red circles.

**Figure S4.** Two dimensions map of thyroid cancer patients urine proteome profiles plotted with MAProMa software. Proteins are plotted according to their theoretical pI and MW (in Log scale). Protein with 1 PSM are reported as yellow triangles, those with PSMs between 2 and 4 as blue squares, and those with a number of PSMs over or equal to 5 as red circles.

**Figure S5.** Venn diagram of proteins distribution among the three groups: A) healthy subjects, B) HNSCC patients, and C) thyroid cancer patients. The area of intersection contained proteins identified in more than one group and the percentage with respect to the total number of proteins identified (365).
