# Supplementary material for: Urinary Proteomics Profiles Are Useful for Detection of Cancer Biomarkers and Changes Induced by Therapeutic Procedures
Source: Molecules. 2019 Feb 22;24(4):794. doi: 10.3390/molecules24040794 (PMC6412696; doi:10.3390/molecules24040794)
Supplement: Supplementary file 1 [file molecules-24-00794-s001.zip › Table S8.docx]

| **Subject** | **Age** | **Diagnosis** | **Study Medication, Dosing** | **Height** | **Weight** | **Body surface area** |
| --- | --- | --- | --- | --- | --- | --- |
| HNSCC | | | | | | |
| P12 | 45,3 | Squamous cell carcinoma of the head and neck region | BPA  8400mg in 280ml | 180cm | 84kg | 2,0m^2^ |
| P21 | 50,6 | Squamous cell carcinoma of the head and neck region | BPA  6000mg in 250ml | 168 | 60kg | 1,7 m^2^ |
| P20 | 68,5 | Squamous cell carcinoma of the head and neck region | BPA  7300mg in 245ml | 172cm | 73kg | 1,9 m^2^ |
| P22 | 53,1 | Squamous cell carcinoma of the head and neck region | BSH  3000mg in 250ml | 168cm | 61kg | 1,7 m^2^ |
| Thyroid carcinoma | | | | | | |
| P11 | 79,6 | Thyroid carcinoma | BSH  2550 in 250ml | 165cm | 51kg | 1,6 m^2^ |
| P14 | 34,6 | Papillary thyroid carcinoma | BSH  4400mg in 250ml | 180cm | 88kg | 2,1m^2^ |
| P16 | 34,0 | Papillary thyroid carcinoma | BPA 1290mg in 430ml | 186cm | 129kg | 2,5m^2^ |

**Table S8.** Subjects analyzed with clinical characteristics.
