# Supplementary material for: Urinary Proteomics Profiles Are Useful for Detection of Cancer Biomarkers and Changes Induced by Therapeutic Procedures
Source: Molecules. 2019 Feb 22;24(4):794. doi: 10.3390/molecules24040794 (PMC6412696; doi:10.3390/molecules24040794)
Supplement: Supplementary file 1 [file molecules-24-00794-s001.zip › Table S7.docx]

| **Term** | **Proteins Count** |
| --- | --- |
| GO:0006898~receptor-mediated endocytosis | 7 |
| GO:0002576~platelet degranulation | 7 |
| GO:0045087~innate immune response | 6 |
| GO:0042742~defense response to bacterium | 5 |
| GO:0006954~inflammatory response | 5 |
| GO:0001895~retina homeostasis | 5 |
| GO:0006508~proteolysis | 5 |
| GO:0006956~complement activation | 5 |
| GO:0006958~complement activation, classical pathway | 5 |
| GO:0010951~negative regulation of endopeptidase activity | 5 |
| GO:0006911~phagocytosis, engulfment | 4 |
| GO:0050853~B cell receptor signaling pathway | 4 |
| GO:0098869~cellular oxidant detoxification | 4 |
| GO:0006910~phagocytosis, recognition | 4 |
| GO:0050871~positive regulation of B cell activation | 4 |
| GO:0038096~Fc-gamma receptor signaling pathway involved in phagocytosis | 4 |
| GO:0007155~cell adhesion | 4 |
| GO:0022617~extracellular matrix disassembly | 3 |
| GO:0006879~cellular iron ion homeostasis | 3 |
| GO:0051180~vitamin transport | 2 |
| GO:0015671~oxygen transport | 2 |
| GO:0061436~establishment of skin barrier | 2 |
| GO:0042744~hydrogen peroxide catabolic process | 2 |
| GO:0010942~positive regulation of cell death | 2 |

**Table S7.** Enrichment in Gene Onthology terms considering Biological processes, performed with DAVID on the differentially expressed proteins lists reported in Table 3 for thyroid cancer patient infused with BSH (after 4 h). The “Proteins count” column shows the number of proteins identified for each GO Biological Process terms.
