# Supplementary material for: Urinary Proteomics Profiles Are Useful for Detection of Cancer Biomarkers and Changes Induced by Therapeutic Procedures
Source: Molecules. 2019 Feb 22;24(4):794. doi: 10.3390/molecules24040794 (PMC6412696; doi:10.3390/molecules24040794)
Supplement: Supplementary file 1 [file molecules-24-00794-s001.zip › Table S6.docx]

| **Protein name** | **Gene Name** | **Accession** | **DAVE**  **After vs before** | **DCI**  **After vs before** |
| --- | --- | --- | --- | --- |
| Histone H2A type 2-A | HIST2H2AA3 | Q6FI13 | 2.00 | 20 |
| Ig gamma-4 chain C region | IGHG4 | P01861 | 2.00 | 44 |
| Keratin, type I cytoskeletal 9 | KRT9 | P35527 | 1.46 | 46 |
| Prostaglandin-H2 D-isomerase | PTGDS | P41222 | 0.51 | 67 |
| Serotransferrin | TF | P02787 | 0.66 | 373 |
| Leucine-rich alpha-2-glycoprotein | LRG1 | P02750 | 0.41 | 20 |
| Protein AMBP | AMBP | P02760 | 0.70 | 148 |
| Ig kappa chain C region | IGKC | P01834 | 0.37 | 193 |
| Hemoglobin subunit beta | HBB | P68871 | 0.34 | 40 |
| Hemopexin | HPX | P02790 | 0.82 | 15 |
| Alpha-1B-glycoprotein | A1BG | P04217 | 0.29 | 12 |
| Keratin, type II cytoskeletal 1 | KRT1 | P04264 | 0.40 | 23 |
| Uromodulin | UMOD | X6RBG4 | -0.25 | -114 |
| Serum albumin | ALB | P02768 | -0.58 | -892 |
| Ceruloplasmin | CP | P00450 | -0.32 | -31 |
| Osteopontin | SPP1 | P10451 | -1.18 | -79 |
| Alpha-1-antitrypsin | SERPINA1 | P01009 | -0.15 | -104 |
| Vitamin D-binding protein | GC | D6RF35 | -0.87 | -31 |
| CD44 antigen | CD44 | P16070 | -1.00 | -11 |
| Apolipoprotein A-I | APOA1 | P02647 | -0.22 | -41 |
| Alpha-1-antichymotrypsin | SERPINA3 | P01011 | -0.46 | -22 |

**Table S6.** DAVE and DCI values of the proteins differentially secreted in urine from a patient affected by thyroid cancer, before and 4 h after the infusion with BSH. The positive values indicate proteins increased after infusion, while negative values indicate proteins decreased after infusion.
