# Supplementary material for: Urinary Proteomics Profiles Are Useful for Detection of Cancer Biomarkers and Changes Induced by Therapeutic Procedures
Source: Molecules. 2019 Feb 22;24(4):794. doi: 10.3390/molecules24040794 (PMC6412696; doi:10.3390/molecules24040794)
Supplement: Supplementary file 1 [file molecules-24-00794-s001.zip › Table S5.docx]

| **Term** | **Proteins Count** |
| --- | --- |
| GO:0002576~platelet degranulation | 7 |
| GO:0045087~innate immune response | 6 |
| GO:0010951~negative regulation of endopeptidase activity | 6 |
| GO:0006898~receptor-mediated endocytosis | 5 |
| GO:0006958~complement activation, classical pathway | 5 |
| GO:0001895~retina homeostasis | 4 |
| GO:0006508~proteolysis | 4 |
| GO:0006956~complement activation | 4 |
| GO:0007155~cell adhesion | 4 |
| GO:0030198~extracellular matrix organization | 4 |
| GO:0038096~Fc-gamma receptor signaling pathway involved in phagocytosis | 3 |
| GO:0001523~retinoid metabolic process | 3 |
| GO:0005975~carbohydrate metabolic process | 3 |
| GO:0006910~phagocytosis, recognition | 2 |
| GO:0050871~positive regulation of B cell activation | 2 |
| GO:2000060~positive regulation of protein ubiquitination involved in ubiquitin-dependent protein catabolic process | 2 |
| GO:0007159~leukocyte cell-cell adhesion | 2 |
| GO:0042572~retinol metabolic process | 2 |

**Table S5.** Enrichment in Gene Onthology terms considering Biological processes, performed with DAVID on the differentially expressed proteins lists reported in Table 2 for HNSCC patient treated with BPA. The “Proteins count” column shows the number of proteins identified for each GO Biological Process terms.
