# Supplementary material for: Urinary Proteomics Profiles Are Useful for Detection of Cancer Biomarkers and Changes Induced by Therapeutic Procedures
Source: Molecules. 2019 Feb 22;24(4):794. doi: 10.3390/molecules24040794 (PMC6412696; doi:10.3390/molecules24040794)
Supplement: Supplementary file 1 [file molecules-24-00794-s001.zip › Table S3.docx]

| **Protein name** | **Gene Name** | **Accession** | **DAVE**  **HSNCC vs TC** | **DCI**  **HSNCC vs TC** | **Fold Change**  **HNSCC vs TC** | **G test HNSCC vs TC** |
| --- | --- | --- | --- | --- | --- | --- |
| Alpha-1-antitrypsin | SERPINA1 | A0A024R6I7 | -0.46 | -34 | -0.47 | 0.9* |
| Cadherin-1 | CDH1 | A0A087WXI5 | -0.67 | -10 | -0.70 | 0.9* |
| ITIH4 protein | ITIH4 | B7ZKJ8 | 0.52 | 27 | 0.53 | 1.0* |
| Alpha-1-antitrypsin | SERPINA1 | P01009 | -0.56 | -49 | -0.58 | 1.5* |
| Alpha-1-antichymotrypsin | SERPINA3 | P01011 | -0.98 | -13 | -1.08 | 1.9* |
| Kininogen-1 | KNG1 | P01042 | -0.80 | -17 | -0.84 | 1.5* |
| Apolipoprotein A-I | APOA1 | P02647 | -1.81 | -13 | -3.02 | 5.4 |
| Retinol-binding protein 4 | RBP4 | P02753 | 2.00 | 18 | 100.00 | 8.3 |
| Protein AMBP | AMBP | P02760 | 0.76 | 137 | 0.80 | 4.0 |
| Alpha-1-acid glycoprotein 1 | ORM1 | P02763 | -1.15 | -10 | -1.32 | 2.1* |
| Serum albumin | ALB | P02768 | -1.11 | -676 | -1.25 | 16.1 |
| Serotransferrin | TF | P02787 | -0.81 | -14 | -0.86 | 1.4* |
| Keratin, type II cytoskeletal 1 | KRT1 | P04264 | -1.30 | -14 | -1.56 | 3.0* |
| Pancreatic alpha-amylase | AMY2A | P04746 | 1.20 | 34 | 1.39 | 4.1 |
| Lysosomal alpha-glucosidase | GAA | P10253 | 1.22 | 13 | 1.41 | 2.6* |
| Osteopontin | SPP1 | P10451 | -0.43 | -38 | -0.44 | 0.9* |
| Aminopeptidase N | ANPEP | P15144 | 1.92 | 14 | 3.86 | 6.4 |
| Alpha-amylase 2B | AMY2B | P19961 | 0.94 | 15 | 1.02 | 1.8* |
| Zinc-alpha-2-glycoprotein | AZGP1 | P25311 | -0.88 | -36 | -0.94 | 2.6* |
| Vasorin | VASN | Q6EMK4 | 0.59 | 25 | 0.61 | 1.1* |
| Uromodulin | UMOD | X6RBG4 | -0.40 | -318 | -0.40 | 2.3* |

**Table S3.** DAVE and DCI values, fold changes, and G test values of the proteins differentially secreted in urine from thyroid cancer patients versus those from HNSCC patients. The positive values indicate proteins overrepresented in HNSCC patients, while negative values indicate proteins overrepresented in thyroid patients. Fold change is expressed as LN(SpC_HNSCC_/SpC_TC_). For G-test a value > 3.8 is equivalent to a p-value <0.05, while a G test value >11.2 is equivalent to a p-value <0.01 * denotes non-significant changes.
