# Supplementary figures and images for: Urinary Proteomics Profiles Are Useful for Detection of Cancer Biomarkers and Changes Induced by Therapeutic Procedures
Source: Molecules. 2019 Feb 22;24(4):794. doi: 10.3390/molecules24040794 (PMC6412696; doi:10.3390/molecules24040794)

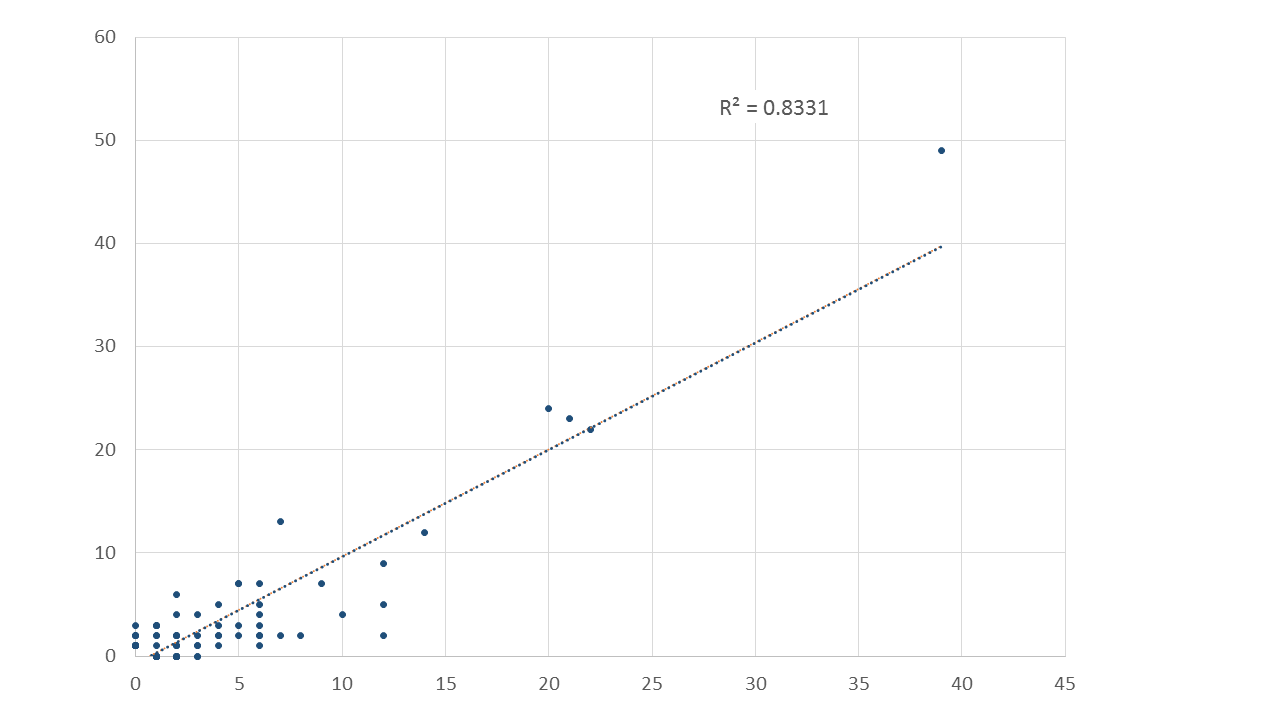

Supplement: Supplementary file 1 [file molecules-24-00794-s001.zip › Figure S1.tif]

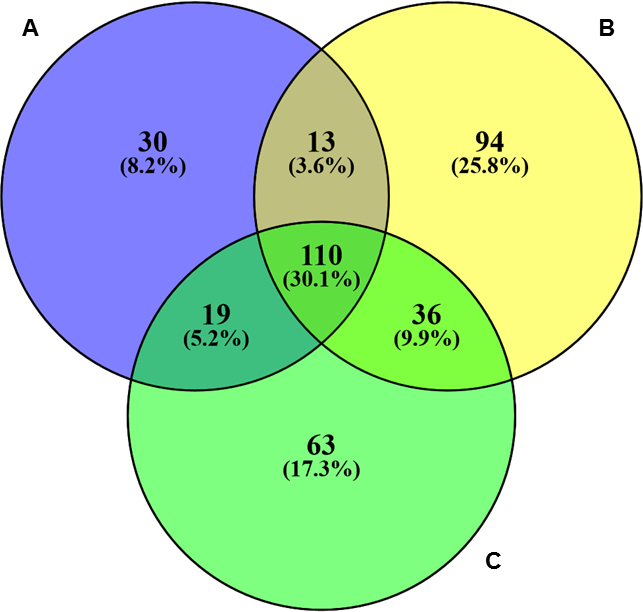

Supplement: Supplementary file 1 [file molecules-24-00794-s001.zip › Figure S5.tif]

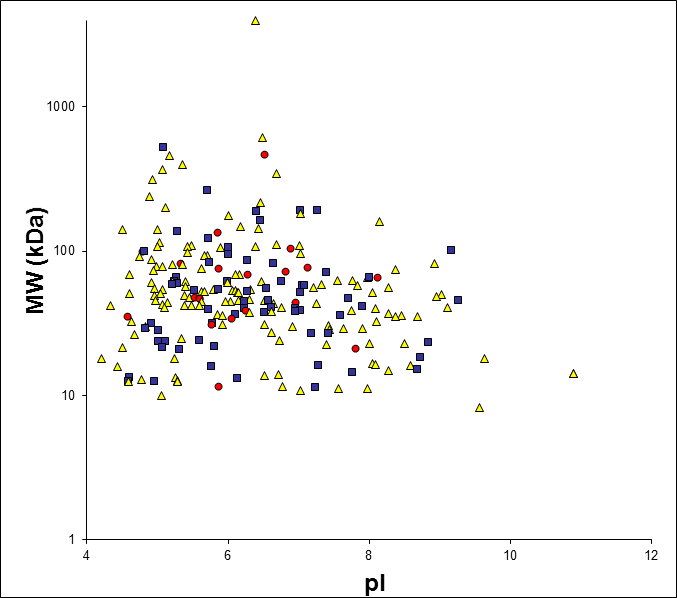

Supplement: Supplementary file 1 [file molecules-24-00794-s001.zip › Figure S4.tif]

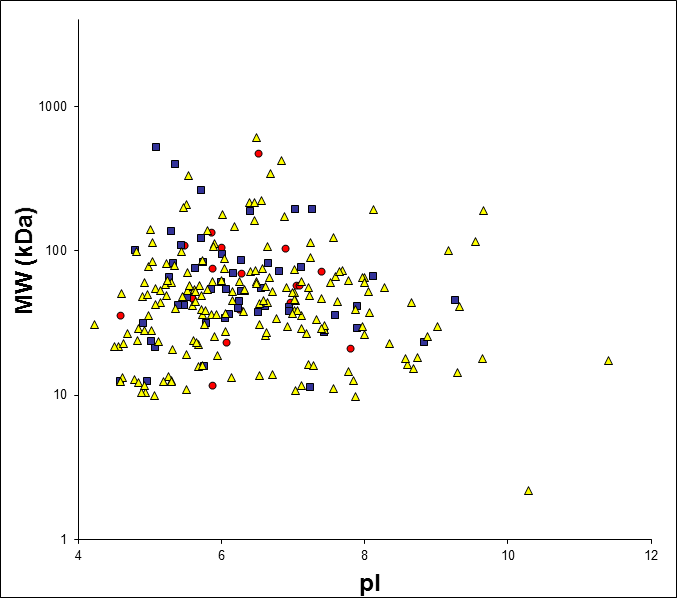

Supplement: Supplementary file 1 [file molecules-24-00794-s001.zip › Figure S3.tif]

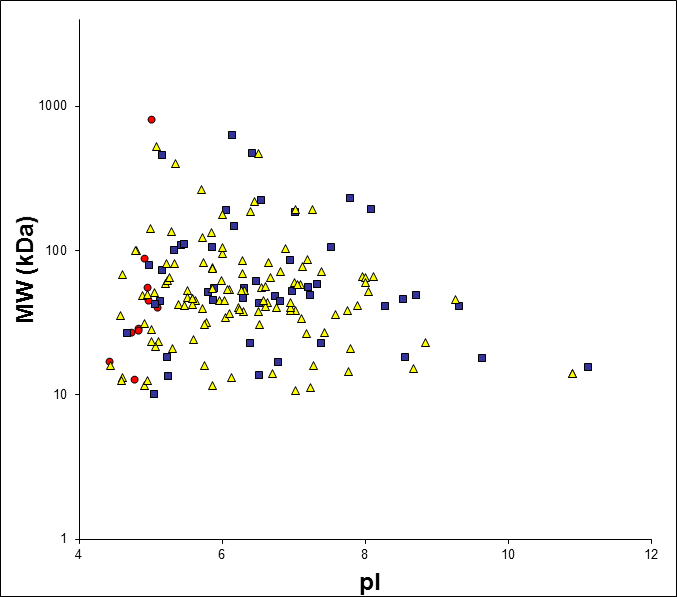

Supplement: Supplementary file 1 [file molecules-24-00794-s001.zip › Figure S2.tif]
